# There Is No Association Between Loiasis and Malaria: Findings from a Secondary Analysis of a Cross-Sectional Survey in Rural Gabon

**DOI:** 10.3390/tropicalmed11020046

**Published:** 2026-02-07

**Authors:** Jacob Werner, Rella Zoleko-Manego, Ghyslain Mombo-Ngoma, Michael Ramharter, Johannes Mischlinger

**Affiliations:** 1 Center for Tropical Medicine, Bernhard Nocht Institute for Tropical Medicine & I. Department of Medicine, University Medical Center Hamburg-Eppendorf, 20359 Hamburg, Germany; 2 German Centre for Infection Research, Partner Site Hamburg-Luebeck-Borstel-Riems, 20359 Hamburg, Germany; 3 Centre de Recherches Médicales de Lambaréné, Lambaréné BP 242, Gabon; 4 Department of Implementation Research, Bernhard Nocht Institute for Tropical Medicine, 20359 Hamburg, Germany

**Keywords:** malaria, loiasis, *Plasmodium* infection, *Loa loa*, co-infection, cross-sectional study, Gabon

## Abstract

Loiasis exists in regions where malaria is highly endemic, yet few studies have investigated their association as concomitant infectious diseases. Secondary data analysis from a cross-sectional survey conducted in Gabon (2015–2016) was performed to assess the association between malaria and loiasis. A total of 947 participants of all ages were enrolled in the original study. In crude analyses, malaria showed a seemingly protective association with loiasis, manifesting in an odds ratio (OR) of 0.67 (95% CI: 0.45 to 1.01; *p* = 0.0521). This borderline association disappeared completely after adjustment for confounders (adjusted OR: 1.31; 95% CI: 0.81 to 2.11; *p* = 0.276), particularly age. The apparent crude protective association is therefore likely explained by the different epidemiological distribution of both diseases according to age rather than a true biological interaction. Malaria predominantly occurred in children and loiasis mainly in older individuals. Findings of this study do not support an association between malaria and loiasis in this setting.

## 1. Introduction

Malaria and loiasis are both clinically and epidemiologically important vector-borne diseases caused by protozoa of the genus *Plasmodium* and the filarial nematode *Loa* (L.) *loa*, respectively [1,2]. While malaria is transmitted by night-biting *Anopheles* mosquitoes that breed in containments of freshwater, *L. loa* is transmitted by day-biting *Chrysops* flies that are associated with forested environments [1,3]. Malaria has long been the focus of intense research, surveillance and control efforts, whereas only recently have reports emerged that indicated a much higher morbidity and mortality of loiasis than had been traditionally assumed, prompting renewed interest in its public health relevance [4]. The geographical distribution of loiasis is largely restricted to parts of Western and Central Africa, areas in which malaria is highly endemic [4,5]. This substantial geographical overlap creates an opportunity for the two infectious diseases to manifest in the same individual or to occur within the same community. In infectious disease research it is well established that certain infections are more or less common in individuals who already harbor another specific infection. A prominent example of such an interaction is the dependence of hepatitis D virus (HDV) infection on pre-existing hepatitis B virus (HBV) carriage [6]. Mechanistically this is explained by the fact that HDV is a defective RNA virus that relies on the hepatitis B surface antigen (HBsAg) for the assembly and envelopment of infectious particles. Comparable, though less prominent, interactions have also been described for malaria, with a potential helminth-induced immune modulation that increases infection susceptibility [7]. In children with *Plasmodium (P.) falciparum* malaria, there is also a well-recognized association with invasive non-typhoidal Salmonella (iNTS) bacteremia, which is believed to be primarily caused by mechanical and immunosuppressive factors arising as part of the given malaria episode which facilitate translocation of NTS from the gut into the bloodstream [8]. Furthermore, evidence suggests a bidirectional interaction between HIV-1 and *P. falciparum* malaria, where co-infection seems to increase infection transmission and worsen the outcomes of both diseases [9]. It is believed that HIV-related immunosuppression increases the risk of acquiring *Plasmodium* infections and increases the severity of malaria episodes, while malaria can raise HIV-1 viral load and may thereby increase HIV transmission risk. Each of these interactions carry important implications for treatment strategies and public health planning. Therefore, it is important to investigate patterns of co-infection and concomitant disease status for co-endemic infectious diseases, such as malaria and loiasis. However, there are only limited published data on this regard. Thus, this short report presents findings on concomitant infectious disease status derived from a large, population-based epidemiological survey conducted in Gabon, a region characterized by intense co-endemicity of both diseases [4,10].

## 2. Materials and Methods

Between December 2015 and February 2016, a large cross-sectional survey on loiasis and malaria was performed at the Centre de Recherches Médicales de Lambaréné (CERMEL) in central Gabon, in the surroundings of Lambaréné and Fougamou [11,12,13,14,15]. After notifying community leaders one week prior, participants were enrolled through active community recruitment. The principal findings of this study were published elsewhere [15]. Here, the results on the concomitant disease status of malaria and loiasis are presented as part of a secondary data analysis. Parasitological analysis was based on the investigation of blood samples, which were sampled around noon. A total of 10 microliters of blood was applied on a glass slide which was stained by 4% Giemsa solution for 60 min. Consequently, microscopists assessed these thick blood smears for the presence of *Plasmodium* species (spp.) and *L. loa* microfilariae. Upon detection of *Plasmodium* spp., effective antimalarial medication was given to the study participant free of charge. Malaria was defined by the presence of *Plasmodium* spp. in a thick blood smear and loiasis was defined by the detection of *L. loa* microfilariae in a thick blood smear or a positive response to RAPLOA (restricted history of eye worm), as described elsewhere [15,16]. The cross-sectional survey received favorable opinions by the local institutional review board and ethics committee (Approval number: 009/2015) [15].

STATA15/SE (StataCorp, College Station, TX, USA) was used for statistical data analysis. Chi-square tests were applied to compare proportions, and Wilcoxon rank-sum tests were used to compare continuous variables with non-parametric distributions. Odds ratios were calculated to quantify associations between binary variables, and logistic regression was used for multivariable analysis. Sex and age were considered a priori confounders and were therefore included in a logistic regression model.

## 3. Results

A total of 947 participants were recruited between December 2015 and February 2016, with a female-to-male ratio of 1.18. The proportion of malaria in the 0–9-year age group was 39.74% (60/151) and decreased progressively in older age groups (Table 1). In contrast, the proportion of loiasis-defining characteristics was low in younger age groups. For example, the proportion of those with a positive case definition for loiasis was 2.42% (7/289) in the 0–9-year age group and increased in older age groups. The proportion of malaria was similar among men and women (*p* = 0.89). On the contrary, *L. loa* microfilaremia was more common in men than in women (56.44% versus 43.56%, respectively; *p* = 0.024), while the proportion of RAPLOA positives was higher among women than men (61.22% versus 38.78%, respectively; *p* = 0.01).

Crude odds ratios indicate that having a loiasis-defining characteristic was associated with decreased odds for malaria (Table 2). Particularly, those harboring *L. loa* microfilaremia had 52% (95% CI: −76% to −1%) lower odds for malaria compared with those negative for *L. loa* microfilaremia. Those with a positive case definition for loiasis had 33% lower odds for malaria (95% CI: −55% to +1%), an association that was borderline significant (0.052). However, after adjusting for the key demographic variables of age and sex, the magnitude of this seemingly protective association not only shifted towards the null value of 1 but also became greater than 1; however, random sampling variation is likely to play a major role in these adjusted associations (*p* > 0.15; Table 2). 

## 4. Discussion

Based on this population-based cross-sectional survey, which included a representative sample of participants living in the target region, a seemingly protective crude association between loiasis and malaria was demonstrated. However, upon closer inspection, this proved to be a false-positive association due to the following reason: in regions of intense malaria transmission, it is a well-described phenomenon that due to the lack of naturally acquired immunity to malaria, young children carry the highest burden of malaria [17]. After surviving several malaria episodes during childhood, semi-immunity gradually develops, thereby making acute malaria episodes less likely as age increases. This is supported by data from this study. While our data confirm existing knowledge that malaria is particularly common in children, loiasis is incrementally more common in older people, resulting from the lack of control programs and repeated infections acquired by living in endemic areas and the long life span of *L. loa* adult worms, which can range up to decades [18]. Consequently, loiasis is more frequently observed among older individuals than in children, simply because they have had longer exposure in endemic areas. Thus, the unadjusted analysis suggests an apparently protective association, with 33% lower odds for loiasis in the presence of malaria and vice versa. However, this is a perfect, classical example of positive confounding, since the presence of loiasis is mostly a proxy for older age and, therefore, the degree of semi-immunity against malaria. This is well illustrated by the fact that when adjusting for age, this apparent protective association shifted towards and beyond the null value of 1 (*p* = 0.276). This finding is educationally interesting, as it underscores the necessity of systematically considering potential confounders, such as age, by inspecting age-specific disease prevalence, performing stratified analyses and including key covariates in multivariable models before inferring causal relationships. Similar false-positive associations are plausible between other long-lived infectious diseases that are geographically co-endemic with malaria, such as onchocerciasis and lymphatic filariasis, where the population prevalence of infection also increases with age.

However, it is important that even such potentially false-positive findings during the first statistical data evaluation prompt further investigation, as it was shown that certain infectious diseases are indeed associated due to various biological reasons. For instance, helminthic infections and malaria show a context-dependent relationship, thought to arise from helminth-induced immune modulation, increasing malaria susceptibility while sometimes limiting inflammatory damage [7,19,20]. In principle, this framework may be extendable to other infections that have an impact on the host’s immune system. These, in turn, may modulate susceptibility to subsequent infections. Furthermore, these primary infections may also influence the clinical severity of those secondary infections [19]. In African pediatric settings it is well established that acute malaria episodes elevate the risk of bacterial infections, particularly non-typhoidal *Salmonella*, and worsen clinical outcomes [8]. The proposed pathways include malaria-driven hemolysis and a transient increase in gut permeability, which enable enteric bacteria to translocate into the bloodstream and amplify the bacteremia risk [21]. Similarly, growing interest has focused on the potential interaction between HIV and malaria in Western sub-Saharan Africa, where the two diseases geographically overlap [9]. A growing body of evidence suggests a bidirectional association between malaria and HIV, in which HIV seems to increase the risk of acquiring malaria while malaria episodes can raise HIV viral load [9]. Yet these interactions are not uniform across the continent, with a stronger interaction observed in the Eastern sub-Saharan African region, where HIV prevalence is high, but a less apparent interaction in the Western sub-Saharan African region, where HIV rates are comparatively low [22]. These are examples of true biological interactions and not merely epidemiological artifacts which can emerge if individuals within a population are differentially likely to be infected based on the presence of another variable (i.e., age in the current case of the seemingly protective association of malaria and loiasis).

It is important to study patterns of co-infection in settings where multiple infectious diseases are co-endemic, particularly because concomitant disease can complicate diagnosis and patient management [2]. For instance, it is a well-described phenomenon that asymptomatic concomitant *Plasmodium* infections can hide the underlying etiology of critically ill individuals if the pathology is attributed to malaria. This can lead to mismanagement of pneumonia, urinary tract infections and ultimately also bacterial bloodstream infections if only malaria treatment is given in such cases [2]. In regions such as Gabon in Central Africa, where both loiasis and malaria are hyperendemic [4,10], clinicians may face patients carrying *Plasmodium* parasites and *L. loa* simultaneously, and it is not always clear whether acute symptoms are driven by malaria, loiasis or their combined effects. This needs to be assessed by further research.

Although age and sex were included as key confounders and age largely explained the apparent association observed in the unadjusted analysis, other potential confounders and sources of bias cannot be ruled out. Important factors, such as living environment, personal protective behavior or prior infection history may also influence the observed relationship. Spatial heterogeneity (e.g., microepidemiological clusters of loiasis overlapping areas of lower local malaria transmission) could potentially have an influence on the observed patterns. Also, the cross-sectional design, a single thick smear and our data collection limited to December–February are acknowledgeable limitations of our study. Nevertheless, in line with the WHO malaria light microscopy guidance, a single thick smear is generally considered sufficiently sensitive in the diagnosis of malaria in endemic settings, mitigating this limitation [23]. Despite these limitations, our study provides an instructive epidemiological example with practical importance for the design and interpretation of large, population-based surveys in regions where loiasis and malaria are co-endemic. Specifically, our findings suggest that there is no apparent association between malaria and loiasis in this study sample, and they illustrate how failure to account for demographic factors, such as age structure, can lead to misleading inferences about co-infection patterns—an important educational lesson for interpreting co-infection data in clinical and public health practice.

## 5. Conclusions

In this population-based survey from a region of intense co-endemicity of loiasis and malaria in Gabon, we found no evidence of an association between these two infectious diseases once age was appropriately accounted for in statistical analysis. The initially observed apparent protective relationship appears to be entirely explained by the age-dependent distribution of both diseases rather than by a direct biological antagonism between the two parasitic diseases. The apparent relationship serves as a textbook example of a striking prima facie association between two infectious diseases that disappears once key confounders are accounted for. It highlights the importance of critically evaluating plausible confounders before interpreting apparent causal relationships. Observational studies investigating associations between pathogens should therefore be grounded in a robust confounder framework to disentangle overlapping risk factors and avoid misleading conclusions.

## Figures and Tables

**Table 1:** Baseline characteristics stratified by microfilaremia and RAPLOA.

		Totals (N = 947)	Malaria			*L. loa* Microfilaremia			RAPLOA			Microfilaremia OR RAPLOA		
			Yes (n, %)	No (n, %)	*p*-Value (Chi2-Test)	Yes (n, %)	No (n, %)	*p*-Value (Chi2-Test)	Yes (n, %)	No (n, %)	*p*-Value (Chi2-Test)	Yes (n, %)	No (n, %)	*p*-Value (Chi2-Test)
Age (in years)														
	median (range)	22 (0.1 to 96)	11 (0.3 to 81)	25.5 (0.1 to 96)	<0.001 *	54 (9 to 96)	17 (0.1 to 96)	<0.001 *	52 (5 to 96)	13 (0.1 to 91)	<0.001 *	51 (5 to 96)	12 (0.1 to 91)	<0.001 *
	0–9	277 (29.25%)	60 (21.66%)	217 (78.34%)	<0.001	2 (0.72%)	275 (99.28%)	<0.001	5 (1.81%)	272 (98.19%)	<0.001	7 (2.53%)	270 (97.47%)	<0.001
	10–19	174 (18.37%)	45 (25.86%)	129 (74.14%)		6 (3.44%)	168 (96.55%)		20 (11.49%)	154 (88.51%)		26 (14.94%)	148 (85.06%)	
	20–29	90 (9.5%)	11 (12.22%)	79 (87.77%)		15 (16.66%)	75 (83.33%)		25 (27.77%)	65 (72.22%)		33 (36.67%)	57 (63.33%)	
	30–39	76 (8.03%)	7 (9.21%)	69 (90.79%)		11 (14.47%)	65 (85.53%)		26 (34.21%)	50 (65.79%)		33 (43.42%)	43 (56.58%)	
	40–49	82 (8.66%)	11 (13.41%)	71 (86.59%)		9 (10.98%)	73 (89.02%)		35 (42.68%)	47 (57.32%)		41 (50%)	41 (50%)	
	50–59	77 (8.13%)	4 (5.19%)	73 (94.81%)		17 (22.08%)	60 (77.92%)		40 (51.95%)	37 (48.05%)		45 (58.44%)	32 (41.56%)	
	60–69	82 (8.66%)	9 (10.98%)	73 (89.02%)		22 (26.83%)	60 (73.17%)		41 (50%)	41 (50%)		49 (59.76%)	33 (40.24%)	
	70–79	54 (5.7%)	3 (5.56%)	51 (94.44%)		7 (12.96%)	47 (87.04%)		32 (59.26%)	22 (40.74%)		32 (59.26%)	22 (40.74%)	
	80–89	32 (3.38%)	1 (3.13%)	31 (96.88%)		11 (34.38%)	21 (65.63%)		19 (59.38%)	13 (40.63%)		21 (65.63%)	11 (34.38%)	
	90–100	3 (0.32%)	0(0%)	3 (100%)		1 (33.33%)	2 (66.67%)		2 (66.67%)	1 (33.33%)		2 (66.67%)	1 (33.33%)	
Sex														
	female	513 (54.17%)	81 (15.79%)	432 (84.21%)	0.89	44 (8.58%)	469 (91.42%)	0.024	150 (29.24%)	363 (70.76%)	0.01	165 (32.16%)	348 (67.84%)	0.23
	male	434 (45.83%)	70 (16.13%)	364 (83.87%)		57 (13.13%)	377 (86.87%)		95 (21.89%)	339 (78.11%)		124 (28.57%)	310 (71.43%)	

* Wilcoxon rank-sum test.

**Table 2:** Associations between malaria and *L. loa* microfilaremia and RAPLOA, respectively.

		Malaria						
		Yes (n, %)	No (n, %)	*p*-Value	Crude ORs (95% CI)	*p*-Value *	Adjusted ORs ** (95% CI)	*p*-Value ***
*L. loa * microfilaremia								
	Not detected	142 (94.04%)	704 (88.44%)	0.041	1		1	
	Detected	9 (5.96%)	92 (11.56%)		0.48 (0.24 to 0.99)	0.041	0.81 (0.38 to 1.7)	0.5672
RAPLOA								
	Negative	120 (79.47%)	582 (73.12%)	0.102	1		1	
	Positive	31 (20.53%)	214 (26.88%)		0.70 (0.46 to 1.08)	0.102	1.4 (0.85 to 2.32)	0.192
*L. loa * microfilaremia OR RAPLOA								
	No	115 (76.16%)	543 (68.22%)	0.052	1		1	
	Yes	36 (23.84%)	253 (31.78%)		0.67 (0.45 to 1.01)	0.0521	1.31 (0.81 to 2.11)	0.276

* H0: RR = 1; ** adjusted for age and sex; *** likelihood ratio test.

## Data Availability

Data are available from the corresponding author upon reasonable request.

## Abbreviations

The following abbreviations are used in this manuscript:

*L. loa*	*Loa loa*
*Plasmodium* spp.	*Plasmodium* species
HIV	Human immunodeficiency virus
